# Comparison of Once-Daily Administration of Edoxaban and Rivaroxaban in Asian Patients with Atrial Fibrillation

**DOI:** 10.1038/s41598-019-43224-4

**Published:** 2019-04-30

**Authors:** So-Ryoung Lee, Eue-Keun Choi, Kyung-Do Han, Jin-Hyung Jung, Seil Oh, Gregory Y. H. Lip

**Affiliations:** 1 Division of Cardiology, Department of Internal Medicine, Seoul National University Hospital, Seoul, Republic of Korea; 2 Department of Medical Statistics, College of Medicine, Catholic University of Korea, Seoul, Republic of Korea; 3 Liverpool Centre for Cardiovascular Science, University of Liverpool and Liverpool Chest & Heart Hospital, Liverpool, United Kingdom; 4 Department of Clinical Medicine, Aalborg University, Aalborg, Denmark

**Keywords:** Cardiology, Drug therapy

## Abstract

It is unclear whether the two once-daily dosing non-vitamin K antagonist oral anticoagulants (NOACs), edoxaban and rivaroxaban, have similar effectiveness and safety in Asian patients with non-valvular atrial fibrillation (AF). This study aimed to compare the effectiveness and safety of edoxaban and rivaroxaban in a Korean population with non-valvular AF. Using the Korean National Health Insurance Service database from January 2014 to December 2016, we compared the risk of ischemic stroke, intracranial hemorrhage (ICH), hospitalization for gastrointestinal (GI) bleeding, hospitalization for major bleeding, all-cause death, and composite outcome in a 3:1 propensity score matched cohort in patients with AF who were naïve to rivaroxaban (n = 12,369) and edoxaban (n = 4,123). Hazard ratios for the six clinical outcomes were analyzed using Cox regression analysis with rivaroxaban as the reference. Baseline characteristics were balanced between the two groups (median age, 71 years; median CHA_2_DS_2_-VASc score, 3; 56% of patients received a reduced dose). Edoxaban users showed comparable results in all six clinical outcomes (all p = nonsignificant) when compared to rivaroxaban users for total, standard, and reduced doses. We provide for the first time the comparison of effectiveness and safety between the two once-daily NOACs in a large-scale Asian AF population. In both standard and reduced dose regimens, edoxaban showed comparable effectiveness and safety compared to rivaroxaban.

## Introduction

For decades, vitamin K antagonists (VKAs) were the only available oral anticoagulants (OACs) for stroke prevention in patients with atrial fibrillation (AF)^1^. Although VKAs are highly effective for the prevention of stroke, their use in patients with AF has been limited by the inconvenience resulting from a narrow therapeutic range and the need for frequent monitoring^2,3^. Furthermore, Asian patients on VKAs are more prone to bleeding, particularly to intracranial hemorrhage (ICH), than non-Asian patients^4,5^.

The recent introduction of non-vitamin K antagonist oral anticoagulants (NOACs) provides effective, safe, and convenient alternatives to VKAs in patients with non-valvular AF^6^. In subgroup analyses of pivotal randomized clinical trials (RCTs), Asians showed greater benefits from NOACs than non-Asians reducing the risk of stroke and ICH, but it is still controversial for risk of gastrointestinal (GI) bleeding^5,7^. In recent observational data from Asians, rivaroxaban showed comparable efficacy results, but increased the risk of hospitalization for GI bleeding compared to warfarin, while edoxaban was associated with a significant lower risk of hospitalization for GI bleeding than warfarin^8,9^. Among four available NOACs, rivaroxaban and edoxaban have the advantage of once-daily administration, allowing convenience and lower pill burden, especially in patients desiring a single dose regimen, thus prescription of these two NOACs has markedly increased^10^. A head-to-head comparison of two treatment could provide useful guidance for physician to choose the most appropriate NOAC for their patients. Therefore, we sought to compare the effectiveness and safety of two once-daily NOACs in the Korean patients with non-valvular AF.

## Methods

The data from the Korean National Health Insurance Service (NHIS) were analyzed. Each patient’s demographic information, International Classification of Disease, Tenth Revision, Clinical Modification (ICD-10-CM) diagnosis codes, procedure codes, and prescription dispending records in inpatient and outpatient services were collected and analyzed. NHIS is a single insurer covering the entire Korean population (approximately 50 million people). Among the enrollees who had a periodic health check-up at least every 2 years, several basic laboratory tests and anthropometric measurements could be obtained. This study was exempted from review by the Seoul National University Hospital Institutional Review Board (E-1802-010-918).

### Study design and cohort definition

**Figure 1:**
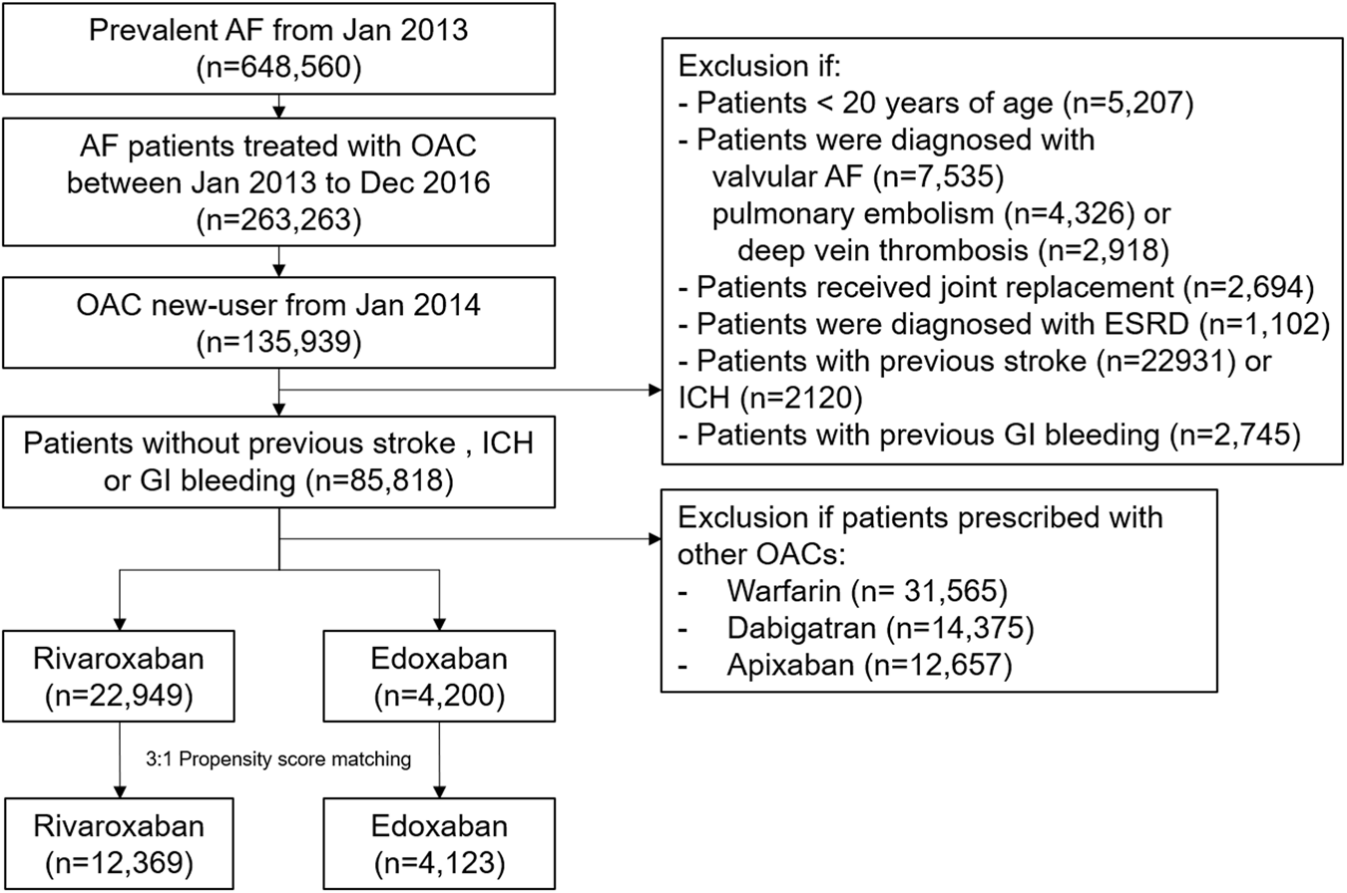
Study population enrollment flow. Abbreviation: AF, atrial fibrillation; ESRD, end-stage renal disease; GI, gastrointestinal; ICH, intracranial hemorrhage; OAC, oral anticoagulants.

Patients who were diagnosed with AF (ICD-10-CM codes I480–I484, I489) between January 2013 and December 2016 were identified. Patients with the following conditions were excluded: (1) mitral stenosis or preexisting mechanical heart valves; (2) previous OAC prescription between January 2013 and December 2013 to analyze only those who were new rivaroxaban and edoxaban users; (3) a potential alternative indication for OAC treatment, such as deep vein thrombosis, pulmonary embolism, or joint replacement; (4) end-stage renal disease; and (5) history of ischemic stroke, ICH, and GI bleeding events. In patients with diagnostic coding of ischemic stroke, ICH, or GI bleeding in the NHIS claims database, incident episodes of those events were not validated to differentiate them from previous episodes^9,11^. The patient enrollment flow is summarized in Fig. 1.

### Baseline covariates

Patients’ baseline characteristics, including age, sex, and comorbidities [hypertension, diabetes mellitus, dyslipidemia, congestive heart failure, peripheral artery disease (PAD), chronic obstructive pulmonary disease (COPD), and prior history of myocardial infarction (MI)], were obtained. Patients’ comorbidities were defined by the ICD-10-CM codes with hospitalization and prescription records within the 1-year period prior to the index date (Supplementary Table 1) and coded as binary variables. CHA_2_DS_2_-VASc score was calculated as a measure of stroke risk in patients with AF by assigning 1 point each for age between 65 and 74 years, female sex, and the presence of hypertension, diabetes mellitus, congestive heart failure, and vascular disease (PAD or prior MI) and adding 2 points each for age of 75 years or older and prior stroke/transient ischemic attack/systemic thromboembolism^12^. The CHADS_2_ score that was used in ROCKET-AF and ENGAGE-AF trials^13,14^, was also calculated as follows: 2 points were assigned for prior stroke/transient ischemic attack and 1 point each was assigned for age ≥75 years, hypertension, diabetes mellitus, or recent congestive heart failure^15^.

### Clinical outcomes and follow-up

The date of the first rivaroxaban or edoxaban prescription during the study period was defined as the index date. To determine the effectiveness and safety of rivaroxaban and edoxaban, six clinical outcomes were identified during study period (between January 2014 and December 2016) as follow: ischemic stroke, ICH, hospitalization for GI bleeding, hospitalization for major bleeding, all-cause death, and composite outcome of ischemic stroke + ICH + all-cause death^9,11^. Clinical outcomes were defined by the ICD-10-CM codes, and detailed definitions are described in Supplementary Table 1. To assess the outcomes, patients were censored at the occurrence of outcome events, or the end of the study period, whichever came first. To balance the follow-up period between two groups, patients were censored at 1 year after index data^9,16^.

### Statistical methods

For the comparison between rivaroxaban and edoxaban-treated groups, a propensity score matching analysis was performed^17,18^. The propensity of being in the rivaroxaban or edoxaban group was estimated with a logistic regression model with all covariates in the baseline characteristics as follows: age, sex, CHA_2_DS_2_-VASc score, hypertension, diabetes mellitus, dyslipidemia, congestive heart failure, prior MI, PAD, and COPD (Supplementary Table 2).

In both rivaroxaban and edoxaban groups, predefined dose reduction criteria were used, because the baseline characteristics of patients taking reduced dose of NOACs (rivaroxaban 15/10 mg and edoxaban 30 mg) might be different from those of patients taking a standard dose of NOACs (rivaroxaban 20 mg and edoxaban 60 mg)^13,14^. For separate analysis by dose regimens, both standard (rivaroxaban 20 mg and edoxaban 60 mg) and reduced dose (rivaroxaban 15/10 mg and edoxaban 30 mg) groups were matched separately based on propensity scores.

Each patient in edoxaban group was matched to three patients in the rivaroxaban group (1:3 matching) because there were more patients who received rivaroxaban than edoxaban (Fig. 1). The greedy, nearest-neighbor method without replacement with a caliper of 0.01 of the propensity scores was used for matching^17^. Baseline characteristics were presented descriptively before and after propensity score matching. Absolute standardized difference (ASD) was used to assess the balance of covariates after matching. An ASD of ≤0.1 (10%) indicated an acceptable difference between the two treatment groups by each covariate^19^.

Crude incidence rates of six clinical outcomes are presented as number of events per 100 person-years (100 PY). The risk of clinical outcomes over time for edoxaban as compared to rivaroxaban (reference) was analyzed suing a survival analysis, with the Kaplan–Meier method and log-rank test for univariate analysis and Cox proportional hazards regression model. Because all baseline covariates were balanced after propensity score matching, the Cox proportional hazards regression included only treatment as the independent variable. Statistical significance was defined as a p value < 0.05. All statistical analyses were performed using SAS version 9.3 software (SAS Institute Inc., Cary, NC, USA).

### Subgroup and sensitivity analyses

Subgroup analyses were performed based on patients’ age, sex, estimated stroke risk, and renal function. Age subgroups were categorized as follows: <65 years, 65–74 years, and ≥75 years. For the estimated stroke risk subgroups, patients were categorized into two groups using CHA_2_DS_2_-VASc scores: 0–2 and ≥3. For the renal function subgroups, patients were categorized into two subgroups by creatinine clearance (CrCl): ≤50 mL/min and >50 mL/min. Subgroup analysis was also performed by patients’ body weight (≤60 kg and >60 kg). Furthermore, patients with CrCl >95 mL/min were analyzed separately to assess the effectiveness and safety of rivaroxaban and edoxaban in patients with “high normal” renal function^20^.

In each subgroup, the balance of baseline characteristics between rivaroxaban and edoxaban groups was evaluated and the covariates with ASD >0.1 (10%) were included in the Cox proportional hazards model. In each subgroup analysis, the interaction between the two treatment modalities in the specific subgroups was evaluated and the statistical significance of the interaction was defined as a p value for interaction <0.1.

A sensitivity analysis was conducted with restriction of the follow-up duration to 6 months because of the shorter follow-up duration of the edoxaban group. Furthermore, we also performed a sensitivity analysis among patients who were only enrolled after February 2016, when edoxaban was introduced into the market^9^.

## Results

During a median of 0.8 years of follow-up [interquartile range (IQR) 0.3–0.9 years)], a total of 27,149 patients with AF newly initiated rivaroxaban (n = 22,949) and edoxaban (n = 4,200). Before propensity score matching, rivaroxaban users had significantly higher CHADS_2_ and CHA_2_DS_2_-VASc scores, and a higher likelihood of hypertension and heart failure compared to edoxaban users (Table 1). Before matching, 51.2% of rivaroxaban users and 56.8% of edoxaban users were prescribed reduced dose regimens. Because of different indications for the standard and reduced doses, the baseline characteristics between the two groups were significantly different in each NOAC group (Supplementary Table 3). Patients taking a reduced NOAC dose were significantly older, more likely to be female, and had higher CHA_2_DS_2_-VASc and CHADS_2_ scores in each NOAC group (Supplementary Table 3).

**Table 1:** Baseline characteristics before and after propensity score matching by treatment group.

	Before propensity score matching			After propensity score matching		
	Rivaroxaban (n = 22,949)	Edoxaban (n = 4,200)	ASD	Rivaroxaban (n = 12,369)	Edoxaban (n = 4,123)	ASD
Age, years						
Mean ± SD	71.5 ± 10.0	70.8 ± 10.0	0.067	71.1 ± 10.0	70.8 ± 10.0	0.037
Median (IQR)	73 (66–78)	72 (65–78)		72 (65–78)	72 (65–78)	
<65	4,899 (21.4)	992 (23.6)		2,850 (23.0)	977 (23.7)	
65–74	8,584 (37.4)	1,606 (38.2)		4,622 (37.4)	1,570 (38.1)	
≥75	9,466 (41.3)	1,602 (38.1)		4,641 (38.1)	1,467 (36.1)	
Men	12,271 (53.5)	2,271 (54.1)	0.473	6,770 (54.7)	2,270 (55.1)	0.006
CHA_2_DS_2_-VASc score						
Mean ± SD	3.62 ± 1.68	3.24 ± 1.62	0.231	3.27 ± 1.61	3.26 ± 1.63	0.006
Median (IQR)	4 (2–5)	3 (2–4)		3 (2–4)	3 (2–4)	
0–1	2,103 (9.2)	561 (13.4)		1,601 (12.9)	549 (13.3)	
2–3	9,171 (40.0)	1,885 (44.9)		5,468 (44.2)	1,826 (44.3)	
≥4	11,675 (50.9)	1,754 (41.8)		5,300 (42.9)	1,748 (42.4)	
CHADS_2_ score						
Mean ± SD	1.93 ± 1.22	1.63 ± 1.16	0.247	1.69 ± 1.15	1.65 ± 1.16	0.034
Median (IQR)	2 (1–3)	2 (1–2)		2 (1–2)	2 (1–2)	
Hypertension	16,740 (72.9)	2,824 (67.2)	0.125	8,632 (69.8)	2,824 (68.5)	0.028
Diabetes mellitus	5,415 (23.6)	845 (20.1)	0.084	2,477 (20.0)	845 (20.5)	0.012
Dyslipidemia	9,611 (41.9)	1,660 (39.5)	0.048	4,679 (37.8)	1,654 (40.1)	0.047
Heart failure	7,320 (31.9)	948 (22.6)	0.211	2,846 (23.0)	948 (23.0)	0.004
Prior MI	775 (3.4)	97 (2.3)	0.064	251 (2.0)	97 (2.4)	0.022
PAD	4,109 (17.9)	710 (16.9)	0.026	1,940 (15.7)	697 (16.9)	0.033
COPD	4,618 (20.1)	748 (17.8)	0.059	2,012 (16.3)	738 (17.9)	0.043

Categorical variables, n (%).

Abbreviation: ASD, absolute standardized difference; COPD, chronic obstructive pulmonary disease; IQR, interquartile range; MI, myocardial infarction; PAD, peripheral artery disease; SD, standard deviation.

### Characteristics of study population in the propensity score-matched cohort

After 3:1 propensity score matching, 12,369 rivaroxaban-treated patients were successfully matched to 4,123 edoxaban-treated patients (Fig. 1). The mean age was 71 ± 10 years (median 72 years, IQR 65–78 years) and mean CHA_2_DS_2_-VASc score was 3.3 ± 1.6 (median 3, IQR 2–4). In matched cohorts, 56% of patients received a reduced NOAC dose (rivaroxaban 15/10 mg and edoxaban 30 mg). Overall, the two matched cohorts were well balanced across all covariates (Table 1, Supplementary Table 4 and Fig. 1). The median follow-up duration was 0.8 years (IQR 0.4–0.9 years) in the rivaroxaban cohort and 0.3 years (IQR 0.1–0.5 years) in the edoxaban cohort (p < 0.001).

### Ischemic stroke, intracranial hemorrhage, hospitalization for gastrointestinal bleeding, hospitalization for major bleeding, all-cause death and composite outcome

**Figure 2:**
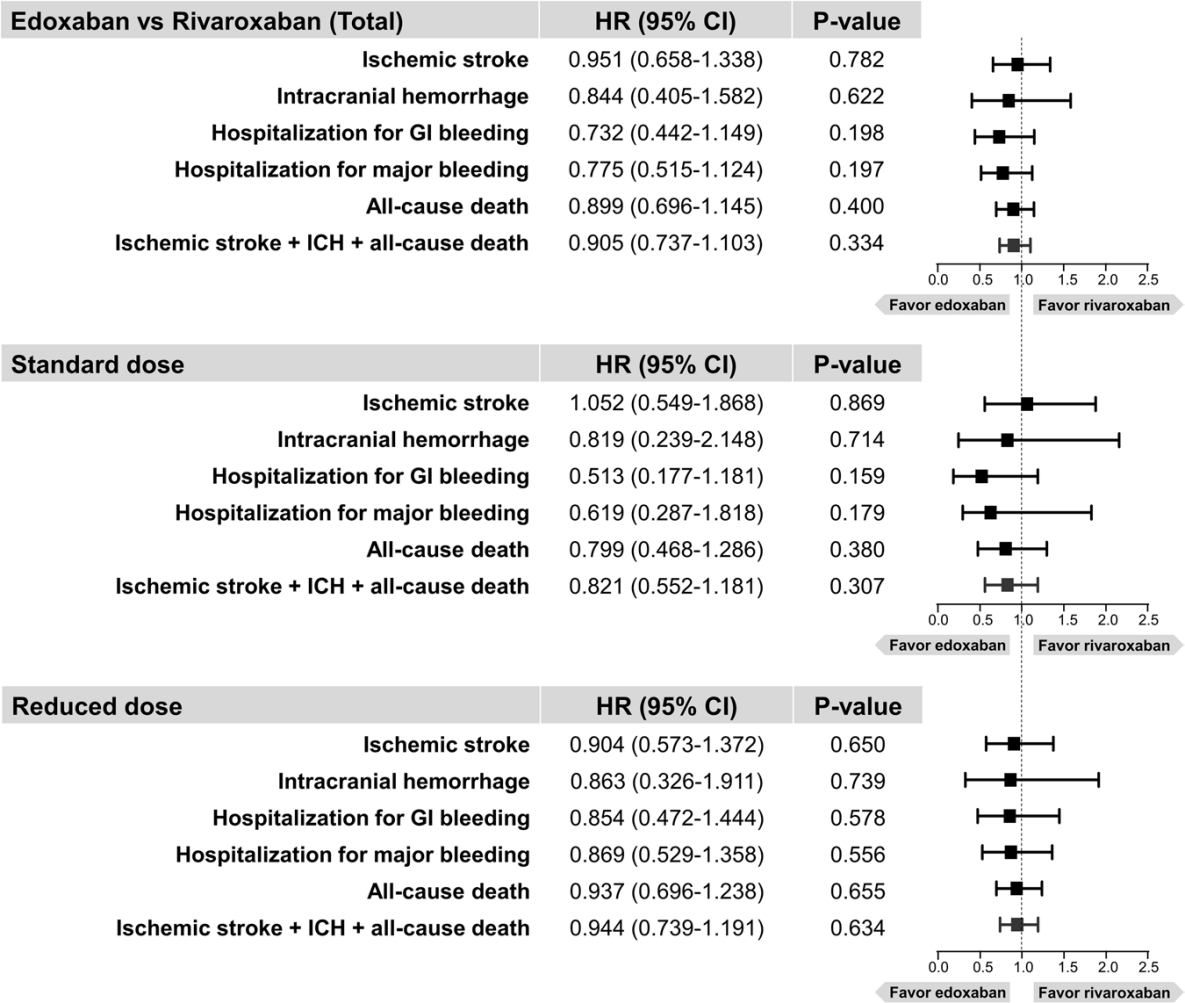
Hazard ratios of six clinical outcomes in rivaroxaban versus edoxaban groups. Abbreviation: CI, confidence interval; GI, gastrointestinal; HR, hazard ratio; ICH, intracranial hemorrhage.

**Figure 3:**
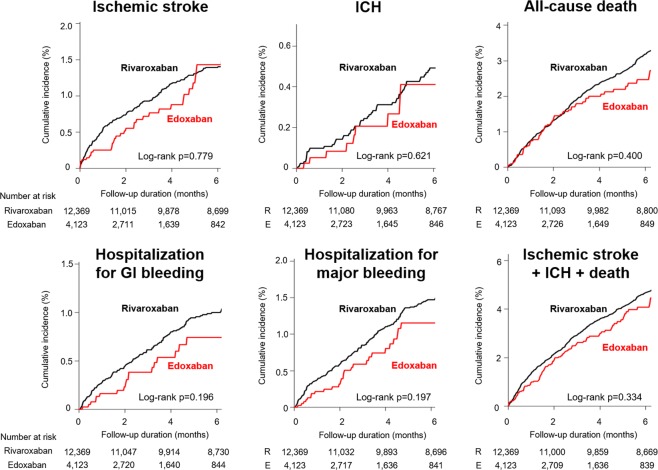
Cumulative incidence of six clinical outcomes in rivaroxaban and edoxaban groups. Abbreviation: GI, gastrointestinal; ICH, intracranial hemorrhage.

Hazard ratios (HRs) of edoxaban treatment with rivaroxaban as the reference were presented in Fig. 2. No significant differences were found between edoxaban and rivaroxaban for all six clinical outcomes (Fig. 2). For hospitalization for GI bleeding, a trend favoring edoxaban compared to rivaroxaban was detected without statistical significance (HR 0.775, 95% CI 0.515–1.124, p = 0.197). Detailed data for the number of events and crude incidence rates according to treatment are summarized in Table 2. The cumulative incidence curves for six clinical outcomes are shown in Fig. 3.

**Table 2:** Number of patients with event and crude incidence rates of six study outcomes.

	Total R versus E		Standard dose		Reduced dose	
	Number of events (IR*)		Number of events (IR*)		Number of events (IR*)	
	R (n = 12,369)	E (n = 4,123)	R 20 mg (n = 5,445)	E 60 mg (n = 1,815)	R 15/10 mg (n = 6,924)	E 30 mg (n = 2,308)
Ischemic stroke	226 (2.76)	38 (3.10)	74 (1.96)	13 (2.34)	152 (3.45)	25 (3.74)
ICH	80 (0.97)	10 (0.81)	31 (0.82)	4 (0.72)	49 (1.10)	6 (0.89)
Hospitalization for GI bleeding	152 (1.85)	20 (1.63)	54 (1.43)	5 (0.90)	98 (2.21)	15 (2.24)
Hospitalization for major bleeding	228 (2.79)	30 (2.45)	84 (2.23)	9 (1.62)	144 (3.26)	21 (3.14)
All-cause death	490 (5.93)	75 (6.09)	136 (3.58)	18 (3.23)	354 (7.93)	57 (8.47)
Ischemic stroke + ICH + all-cause death	722 (8.86)	115 (9.41)	225 (5.99)	32 (5.77)	497 (11.30)	83 (12.43)

*IR, per 100 person-years.

Abbreviation: CI, confidence interval; E, edoxaban; GI, gastrointestinal; HR, hazard ratio; ICH, intracranial hemorrhage; IR, incidence rate; R, rivaroxaban.

### Sensitivity analyses

**Figure 4:**
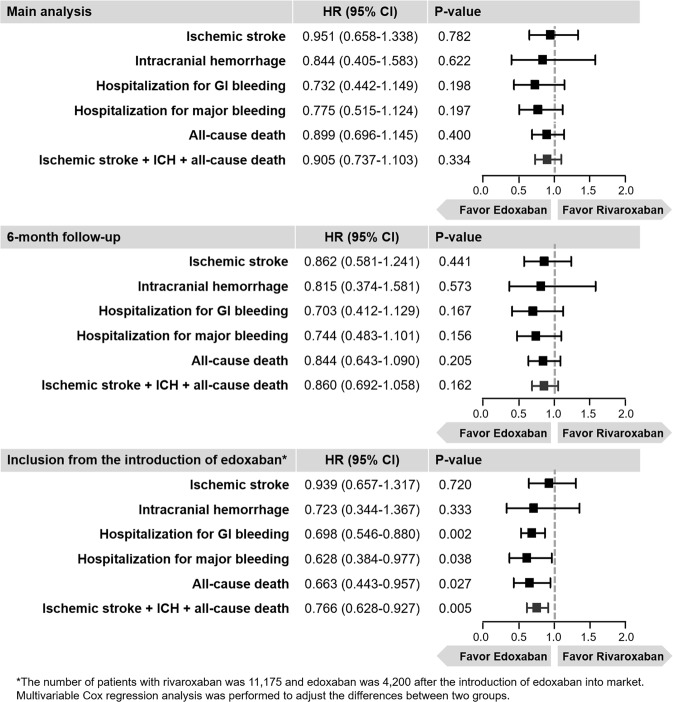
Sensitivity analyses restricting the follow-up duration to 6-month and from same period. Abbreviation: CI, confidence interval; GI, gastrointestinal; HR, hazard ratio; ICH, intracranial hemorrhage.

In sensitivity analyses for adjusting the differences in follow-up duration to 6 months, HR trends for all clinical outcomes were similar to the main results (Fig. 4). While comparing those with same period of enrollment, edoxaban showed lower risks of hospitalization for GI bleeding (HR 0.698, 95% CI 0.546–0.880, p = 0.002), hospitalization for major bleeding (HR 0.628, 95% CI 0.384–0.977, p = 0.038), all-cause death (HR 0.663, 95% CI 0.443–0.957, p = 0.027), and composite outcomes (HR 0.766, 95% CI 0.628–0.927, p = 0.005) compared to rivaroxaban (Fig. 4).

### Outcomes according to dose regimens

**Figure 5:**
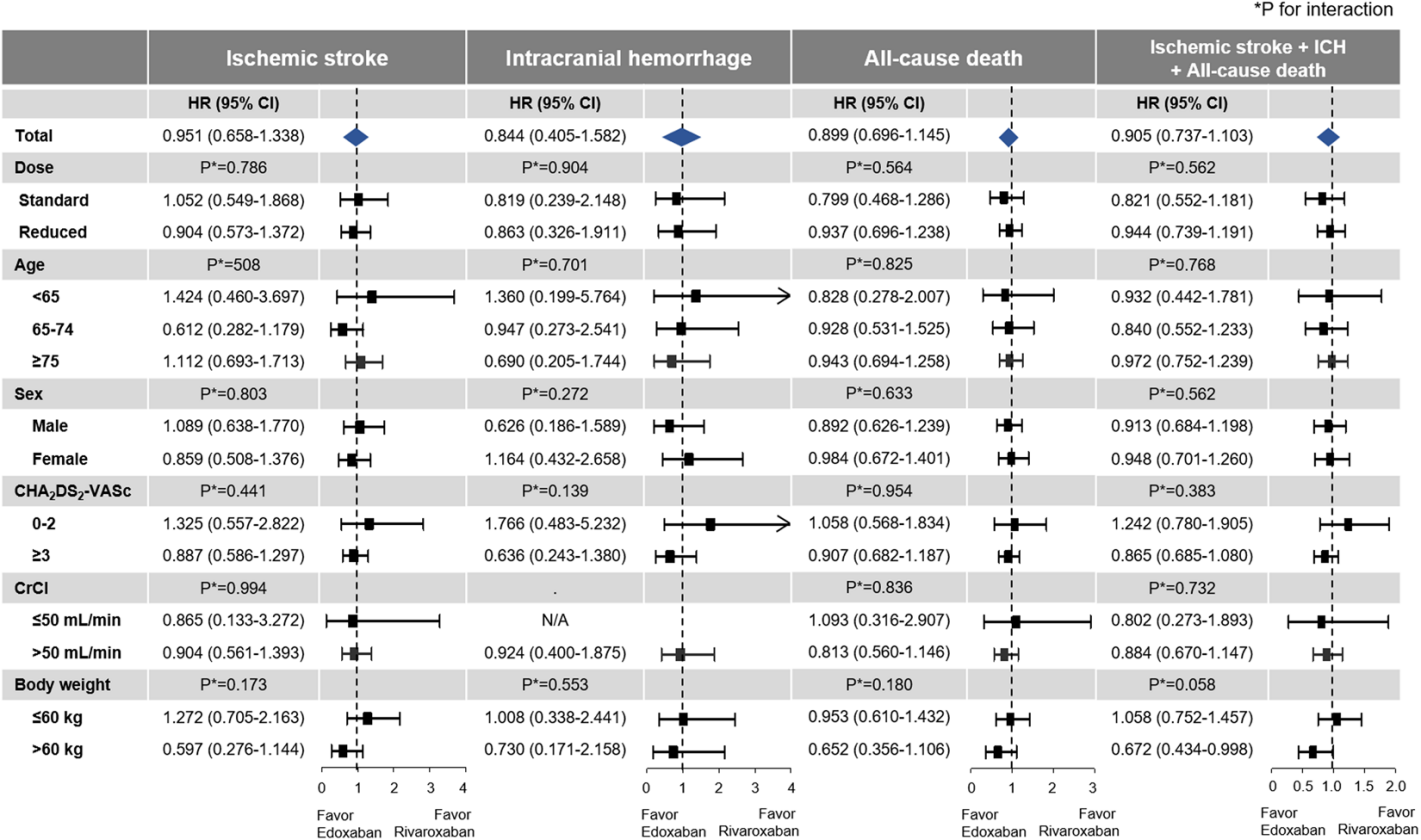
Hazard ratios for ischemic stroke, intracranial hemorrhage, all-cause death, and composite outcome according to subgroups in rivaroxaban and edoxaban groups. Abbreviation: CI, confidence interval; CrCl, creatinine clearance; HR, hazard ratio; ICH, intracranial hemorrhage.

**Figure 6:**
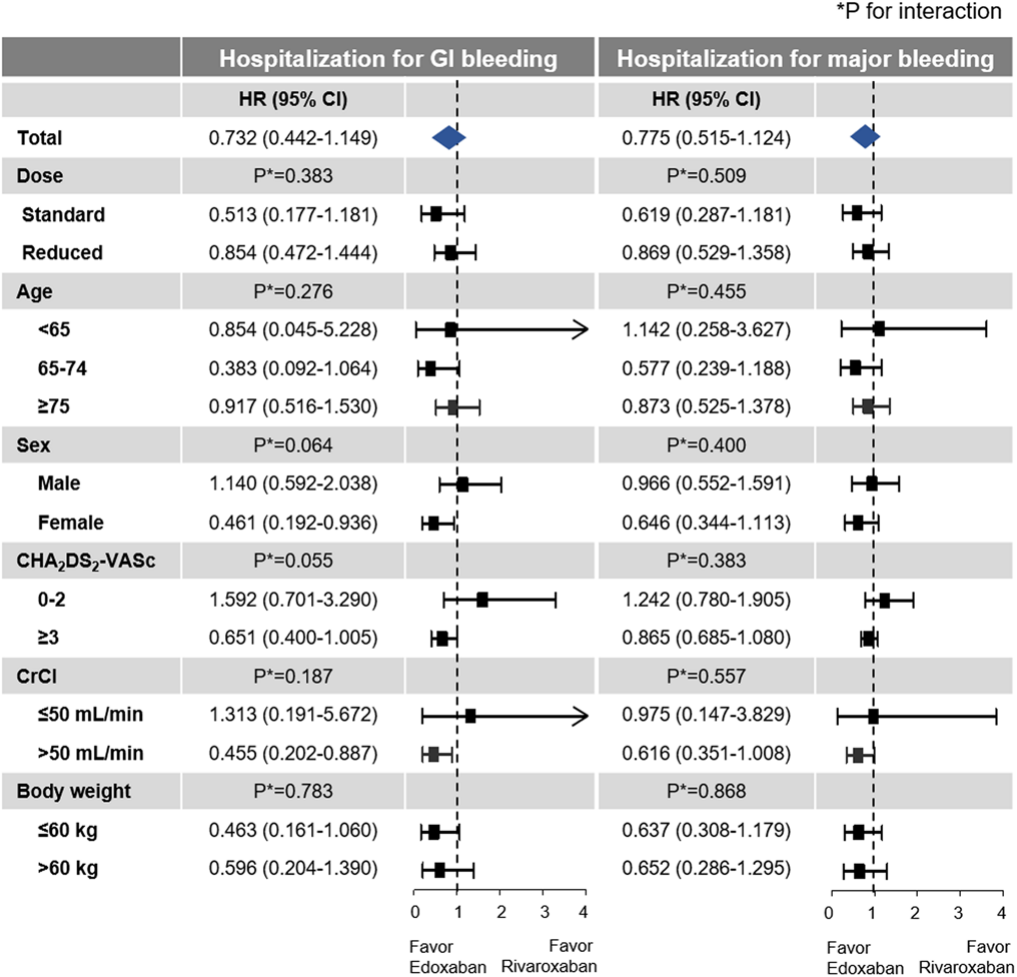
Hazard ratios for hospitalization for gastrointestinal bleeding and hospitalization for major bleeding according to subgroups in rivaroxaban and edoxaban groups. Abbreviation: CI, confidence interval; CrCl, creatinine clearance; GI, gastrointestinal; HR, hazard ratio.

The cumulative incidences of six clinical outcomes are shown in Supplementary Figs 2 and 3. Compared to the standard dose rivaroxaban group, patients receiving the standard dose edoxaban had similar risks of ischemic stroke and ICH (HR 0.819, 95% CI 0.239–2.148, p = 0.714) (Fig. 2). Among patients taking the reduced dose regimen, both rivaroxaban and edoxaban showed similar risks for all six clinical outcomes (Fig. 2). HRs of edoxaban compared to rivaroxaban were generally consistent in both standard and reduced dose regimens. No significant interaction was found between treatment and dose regimens in all six clinical outcomes (Figs 5 and 6). Detailed data for the number of events and crude incidence rates according to treatment by dose regimens are summarized in Table 2.

### Subgroup analysis

HRs were generally consistent among subgroups (Figs 5 and 6 and Supplementary Table 5). Ischemic stroke, ICH, hospitalization for GI bleeding, hospitalization for major bleeding, all-cause death, and composite outcome were all consistent across the three age subgroups, for rivaroxaban and edoxaban, with no significant interaction detected. No significant interaction was found between treatment and sex in regard to all six clinical outcomes.

Across the lower and higher stroke risks groups (CHA_2_DS_2_-VASc score 0–2 and ≥3), no significant difference was found between rivaroxaban and edoxaban in six clinical outcomes.

Among patients with available CrCl value (80% of population in each treatment group), 654 (5%) patients had moderate renal dysfunction (CrCl ≤50 mL/min). Edoxaban and rivaroxaban showed generally comparable outcomes in both groups stratified by renal function (Figs 5 and 6). Although there was no significant interaction between treatment and renal function, edoxaban was associated with lower risk of hospitalization for GI bleeding compared to rivaroxaban in patients with CrCl >50 mL/min (HR 0.455, 95% CI 0.202–0.887, p = 0.034). The incidence of ischemic stroke was also not significantly different between the edoxaban and rivaroxaban users (2.21 vs. 2.41 per 100 PY) in patients with “high normal” renal function (CrCl >95 mL/min). In patients with “high normal” renal function, edoxaban use did not increase the risk of ischemic stroke compared to rivaroxaban use (HR, 0.706; 95% CI 0.205–1.858; p = 0.524).

Edoxaban and rivaroxaban showed generally comparable outcomes in both groups stratified by body weight, except for composite outcome (Figs 5 and 6). Edoxaban showed better composite outcome than rivaroxaban in patients with body weight >60 kg, of borderline significance (HR 0.672, 95% CI 0.434–0.998, p = 0.049, p for interaction = 0.058).

## Discussion

To our knowledge, this is the first head-to-head comparison of the effectiveness and safety of the two once-daily NOACs (rivaroxaban and edoxaban) in a large-scale Asian population with non-valvular AF. We demonstrated that rivaroxaban and edoxaban use was associated with similar risks of ischemic stroke, ICH, all-cause death and the composite outcome.

Our data showed that both once-daily NOAC regimens were associated with similarly well results in “real-world” clinical practice. Both showed similar efficacy and safety compared to warfarin in large randomized clinical trials (RCTs)^13,14^. In the ROCKET-AF trial^13^, rivaroxaban (n = 7,131) was non-inferior to warfarin for stroke/systemic embolism prevention and showed a similar risk of major and clinically relevant non-major bleeding. Although rivaroxaban was associated with a lower risk of ICH (0.5% vs. 0.7%, p = 0.02), major GI bleeding was more frequent in the rivaroxaban group than in the warfarin group (3.2% vs. 2.2%, p < 0.001). In the ENGAGE-AF trial^14^, high dose edoxaban regimen (HDER, 60/30 mg arm, n = 7,035) was noninferior for stroke/systemic embolism compared to warfarin, but showed a reduced risk of major bleeding, mainly driven by a reduction in ICH. Edoxaban significantly reduced ICH by 53%, but increased major GI bleeding by 23% compared to warfarin.

Despite the reassuring data from these pivotal RCTs evaluating the efficacy and safety of rivaroxaban and edoxaban compared to warfarin, further investigation is needed given that the baseline characteristics of two studies were different. Patients enrolled in the ROCKET-AF tended to have higher CHADS_2_ scores than those included in the ENGAGE-AF trial (mean CHADS_2_ score, 3.5 vs. 2.8, respectively)^13,14^. In addition, the performance of warfarin group reflected by time in therapeutic range (TTR) was different between ROCKET-AF (55%) and ENGAGE-AF (68%). These trial population differences might make the indirect comparison between rivaroxaban and edoxaban by extrapolating from two pivotal clinical trials difficult. Furthermore, these RCTs included a relatively small proportion of Asians (6.5% in ROCKET-AF and 9.2% in ENGAGE-AF), as well as a smaller proportion of patients who received the reduced dose regimen (21% in ROCKET-AF and 25.4% in ENGAGE-AF) when compared with Asian “real-world” clinical settings^8,11^.

Several studies have reported the results of indirect and direct comparisons among three NOACs, including rivaroxaban, dabigatran, and apixaban, based on “real-world” observational databases^8,11,21–23^. Given the results of indirect comparisons using warfarin as a common comparator, the risk of major bleeding with rivaroxaban did not significantly differ from that with warfarin, but resulted in more GI bleeding^21,22^. The results of comparisons with dabigatran or apixaban found that rivaroxaban was associated with a higher risk of major bleeding and GI bleeding^22,23^. When focused on the Asian population, compared to warfarin, rivaroxaban was associated with reduced risks of ischemic stroke, ICH, and all-cause death without significantly increasing the risk for hospitalization for GI bleeding (HR, 1.43; 95% CI, 0.88–2.33) and major bleeding (HR, 0.77; 95% CI, 0.53–1.13)^8^.

In general, data from observational cohorts has provided complementary and consistent evidence on the efficacy and safety of the NOACs to those obtained in pivotal RCTs^24^. Recently, we reported a comparison between edoxaban and warfarin in a large nationwide Asian cohort and found that edoxaban was associated with a lower risk of ischemic stroke, ICH, and all-cause death^9^. Furthermore, edoxaban significantly reduced the risk of hospitalization for GI bleeding and major bleeding in Asian population.

Our results were inconsistent with main results of ENGAGE-AF, but we should consider the significant interaction between non-Asian and Asian in bleeding outcomes^7^ and the minority of Asians included in the RCTs. Of note, in the pooled meta-analysis of 4 pivotal RCTs, the increase risk of GI bleeding was only significant in non-Asian patients and a significant interaction was found between non-Asian and Asian patients^6^.

Given that some patients (and physicians) express a preference for the convenience of once-daily dosing, a recurrent question is whether rivaroxaban is better or comparable to edoxaban in terms of efficacy and safety. In the absence of head-to-head trials, Skjøth *et al*. performed an indirect comparison analysis using the data from the 4 pivotal RCTs and found that rivaroxaban use was associated with significantly more major and/or clinically relevant non-major bleeding and a nonsignificant increase in hospitalization for GI bleeding compared to edoxaban^25^. In line with previous study, we found that the two once-daily dosing NOACs showed overall comparable outcomes, whereas edoxaban tended to be associated with a lower GI bleeding risk compared to rivaroxaban.

In the present well-matched cohort study using nationwide data, the two once-daily dosing NOACs showed comparable outcomes. Consistent with the data of RCTs and previous observational data, edoxaban tended to be associated with a lower GI bleeding risk compared to rivaroxaban, but the difference was not statistically significant. All six outcomes were generally consistent across all subgroups and no significant interaction was found between treatment and specific subgroups, but with some exceptions. Edoxaban was associated with a lower risk of hospitalization for GI bleeding in female patients and in patients with CrCl >50 mL/min. Although the results of subgroup analyses should be carefully interpreted, some differential effects detected in patients with different clinical profile might be useful for physicians to be able to fit the treatment to their patient’s clinical profile.

Reduced dose NOACs are frequently prescribed in the Asian population regardless of label adherence of dosing^8,9,11^. In this study, CrCl and body weight were available in only 80% of patients; thus, label adherence of NOAC dosing could not be fully evaluated. Among patients with available CrCl value and body weight information, 93% on rivaroxaban 15/10 mg and 44% on edoxaban 30 mg received inappropriate dose reduction. In previous studies, prescription of inappropriately reduced dose NOACs (off-label NOAC underdosing) was associated with an increased risk of stroke without a benefit in safety^26,27^. However, the clinical implications of off-label NOACs underdosing remains controversial. Asian patients have relatively lower body weight than non-Asian patients and are more prone to bleeding, including ICH with warfarin^5,28^. Hence, some Asian countries have adopted different dosing regimens for rivaroxaban using 15 mg as a standard dose^29^. Although edoxaban and rivaroxaban showed similar trends of HRs in both standard and reduced dose regimens in this study, these data should be cautiously interpreted because reduced dose groups included a substantial portion of patients with off-label underdosing of NOACs.

### Study limitations

Several limitations of this study should be acknowledged. First, despite using several variables and applying advanced statistical methods like propensity score matching to reduce the effect of confounding, we cannot completely eliminate bias from residual confounding by physicians’ treatment decisions and unmeasured factors, which is a general limitation of observational studies. Thus, we could not measure the variables that were not included within the claims database. Second, we used widely used definitions of covariates and clinical outcomes which were also validated in our previous studies^9,11,30–32^. However, there is inherent limitation based on claim data. Third, patients with prior ischemic stroke, ICH, or GI bleeding were excluded from this study. Incident episodes of those particular events were not validated to allow differentiation with previous events in those who had a prior history of ischemic stroke, ICH, or GI bleeding events; therefore, the results of this study could not be extrapolated to those with history of ischemic stroke, ICH, or GI bleeding. Compared to pivotal RCTs, our study population had lower mean CHADS_2_ score (3.5 in ROCKET-AF^13^, 2.8 in ENGAGE-AF^14^ and 1.7 in both rivaroxaban and edoxaban groups in this study). Further study would be needed for multi-morbid and high-risk patients. Fourth, given the more recent introduction of edoxaban in the market, the follow-up period for edoxaban was shorter than rivaroxaban. Although we demonstrated the consistent results after adjusting for the differences in follow-up duration and period between two groups, overall short follow-up duration in both treatment groups could be still a limitation. It is unclear whether more significant divergence in clinical outcomes between two treatment groups after long-term follow-up. Fifth, the cause of death was not available in this dataset, thus we could not provide the HR of cardiovascular and non-cardiovascular death. However, consistent with previous studies based on observational database, we have only reported the results of all-cause death as one of relevant hard end points^8,9,11,21^. Sixth, although there are recently published and on-going trials about NOAC use in patients with AF and special clinical situations such as percutaneous coronary intervention^33,34^ or AF catheter ablation^35,36^, the numbers of patients who underwent procedures in the present study were too small (less than 2%), so further analyses would not be feasible. Finally, the actual drug adherence could not be evaluated owing to the inherent limitation of claims data.

Despite these limitations, overall, rivaroxaban and edoxaban showed comparable results in effectiveness and safety from this study. There has been lack of information about the effectiveness and safety of edoxaban, lastly introduced NOAC, compared to rivaroxaban, especially in “real-world setting in Asians”. Based on our study results, edoxaban could be a good treatment option for patients who want lower pill burden, as well as being preferred in patients concerned about GI bleeding events when considering two possible options of once-daily dosing NOACs. Once-daily regimens could be more convenient than twice-daily regimens; therefore, higher medication adherence would be expected^37^. Theoretically, the clinical impact of a single dose missed might be greater in once-daily dosing than twice-daily dosing^38^. In clinical practice, therefore, once-daily dosing NOACs may require more vigilance for missed doses or non-adherence, and various efforts to improve drug adherence should be implemented^10,39^.

## Conclusions

To our knowledge, this is the first head-to-head comparison of the effectiveness and safety between the two once-daily NOAC regimens (rivaroxaban, edoxaban) in a nationwide Asian cohort with non-valvular AF. In both standard and reduced dose matching cohorts, edoxaban and rivaroxaban were associated with similar outcomes for ischemic stroke, ICH, hospitalization for GI bleeding and major bleeding, all-cause death, and the composite outcome.

## Supplementary information

Supplementary Materials
