# Supplementary material for: Comparison of Once-Daily Administration of Edoxaban and Rivaroxaban in Asian Patients with Atrial Fibrillation
Source: Sci Rep. 2019 Apr 30;9:6690. doi: 10.1038/s41598-019-43224-4 (PMC6491445; doi:10.1038/s41598-019-43224-4)
Supplement: Supplementary file 1 — Supplementary Materials [file 41598_2019_43224_MOESM1_ESM.docx]

**Comparison of Once-Daily Administration of Edoxaban and Rivaroxaban in Asian Patients with Atrial Fibrillation**

So-Ryoung Lee,^a^ Eue-Keun Choi,^a^* Kyung-Do Han,^b^ Jin-Hyung Jung,^b^ Seil Oh,^a^ Gregory Y H Lip^c,d^

^a^Division of Cardiology, Department of Internal Medicine, Seoul National University Hospital, Seoul, Republic of Korea

^b^Department of Medical Statistics, College of Medicine, Catholic University of Korea, Seoul, Republic of Korea

^c^ Liverpool Centre for Cardiovascular Science, University of Liverpool and Liverpool Chest & Heart Hospital, Liverpool, United Kingdom; and ^d^ Department of Clinical Medicine, Aalborg University, Aalborg, Denmark

**Online Data Supplements**

**Contents of the Supplementary Materials**

**I. Supplementary Tables**

**II. Supplementary Figures**

**I. Supplementary Tables**

**Table S1. Definition of comorbidities and clinical outcomes**

| **Diagnosis** | **ICD-10-CM code and definition** | **Diagnostic definition** |
| --- | --- | --- |
| **Atrial fibrillation** | I480-484, I489 | Admission or outpatient department≥1 |
| **Valvular atrial fibrillation** | I050, I052, I059, Z952-Z954 |  |
| **Pulmonary embolism** | I26 | Admission≥1 |
| **Deep vein thrombosis** | I802 | Admission≥1 |
| **Received joint replacement operation** | N0711, N1711, N1721, N2070, N3710, N3721, N3717, N3720, N2072, N2077, N3722, N3727 | Admission≥1 |
| **End stage renal disease** | N185, Z49 | Dialysis≥2 |
| **Ischemic stroke** | I63, I64 | Admission≥1 and brain imaging (CT or MRI) ≥1 |
| **Intracranial hemorrhage** | I60-62 | Admission≥1 or RBC transfusion≥1 |
| **Hospitalization for GI bleeding** | K22.6, K25.0, K25.2, K25.4, K25.6, K26.0, K26.2, K26.4, K26.6, K27.0, K27.2, K27.4, K27.6, K28.0, K28.2, K28.4, K28.6, K29.0, K62.5, K92.0, K92.1, K92.2 | Admission≥1 and RBC transfusion≥1 |
| **Hospitalization for major bleeding** | Intracranial bleeding or gastrointestinal bleeding | ICH, admission≥1 or RBC transfusion≥1  GI bleeding, admission≥1 and RBC transfusion≥1 |
| **Hypertension** | I10-I13, I15; and minimum 1 prescription of anti-hypertensive drug (thiazide, loop diuretics, aldosterone antagonist, alpha-/beta-blocker, calcium-channel blocker, angiotensin-converting enzyme inhibitor, angiotensin II receptor blocker). | Admission≥1 or outpatient department≥2 |
| **Diabetes mellitus** | E11-E14; and minimum 1 prescription of anti-diabetic drugs (sulfonylureas, metformin, meglitinides, thiazolidinediones, dipeptidyl peptidase-4 inhibitors, α-glucosidase inhibitors and insulin). | Admission≥1 or outpatient department≥2 |
| **Dyslipidemia** | E78 | Admission or outpatient department≥1 |
| **Heart failure** | I50 | Admission or outpatient department≥1 |
| **Vascular disease** |  |  |
| **Prior MI** | I21, I22 | Admission or outpatient department≥1 |
| **PAD** | I70, I73 | Admission or outpatient department≥2 |
| **COPD** | J41-44 | Admission or outpatient department≥1 |

Abbreviation: COPD, chronic obstructive pulmonary disease; GI, gastrointestinal; MI, myocardial infarction; PAD, peripheral artery disease.

**Table S2. The logistic regression model used to calculate the propensity score**

|  | **Coefficient** | **SE** | **OR (95% CI)** | **P value** |
| --- | --- | --- | --- | --- |
| **Reduced dose matching** |  |  |  |  |
| **Age (per year)** | 0.009 | 0.003 | 1.009 (1.003-1.016) | 0.004 |
| **Sex (male)** | -0.142 | 0.027 | 0.753 (0.678-0.835) | <0.001 |
| **CHA_2_DS_2_-VASc score (per point)** | -0.124 | 0.027 | 0.883 (0.839-0.931) | <0.001 |
| **Diabetes mellitus** | 0.029 | 0.031 | 1.060 (0.939-1.196) | 0.3484 |
| **Hypertension** | 0.104 | 0.027 | 1.232 (1.110-1.367) | <0.001 |
| **Dyslipidemia** | 0.019 | 0.024 | 1.039 (0.944-1.143) | 0.434 |
| **Congestive heart failure** | 0.098 | 0.031 | 1.218 (1.080-1.373) | 0.001 |
| **Prior MI** | 0.044 | 0.072 | 1.092 (0.824-1.448) | 0.538 |
| **COPD** | 0.001 | 0.028 | 1.002 (0.897-1.120) | 0.969 |
| **PAD** | -0.088 | 0.033 | 0.838 (0.736-0.955) | 0.008 |
| **Standard dose matching** |  |  |  |  |
| **Age (per year)** | -0.003 | 0.003 | 0.997 (0.990-1.003) | 0.328 |
| **Sex (male)** | 0.004 | 0.031 | 1.008 (0.892-1.140) | 0.895 |
| **CHA_2_DS_2_-VASc score (per point)** | -0.218 | 0.031 | 0.804 (0.756-0.855) | <0.001 |
| **Diabetes mellitus** | 0.020 | 0.034 | 1.040 (0.910-1.189) | 0.565 |
| **Hypertension** | -0.053 | 0.031 | 0.900 (0.798-1.016) | 0.089 |
| **Dyslipidemia** | -0.059 | 0.027 | 0.888 (0.799-0.987) | 0.028 |
| **Congestive heart failure** | 0.157 | 0.037 | 1.369 (1.186-1.581) | <0.001 |
| **Prior MI** | 0.046 | 0.090 | 1.097 (0.771-1.561) | 0.606 |
| **COPD** | 0.055 | 0.037 | 1.116 (0.967-1.288) | 0.134 |
| **PAD** | -0.103 | 0.039 | 0.815 (0.698-0.951) | 0.009 |

Abbreviation: CI, confidence interval; COPD, chronic obstructive pulmonary disease; MI, myocardial infarction; OR, odds ratio; PAD, peripheral artery disease; SE, standard error of coefficient.

**Table S3. Difference in baseline characteristics between regular and reduced dose in each NOAC**

|  | Rivaroxaban | | |  | Edoxaban | | |
| --- | --- | --- | --- | --- | --- | --- | --- |
|  | Standard  (20mg)  (n=11,200) | Reduced  (15/10mg)  (n=11,749) | ASD |  | Standard  (60mg)  (n=1,815) | Reduced  (30mg)  (n=2,385) | ASD |
| Age, years |  |  |  |  |  |  |  |
| Mean ± SD | 68.9±10.0 | 73.9±9.2 | 0.522 |  | 66.7±9.6 | 74.0±9.2 | 0.773 |
| Median (IQR) | 70 (63-76) | 75 (69-80) |  |  | 68 (61-74) | 75 (69-80) |  |
| <65 | 3,258 (29.1) | 1,641 (14.0) |  |  | 675 (37.2) | 317 (13.3) |  |
| 65-74 | 4,570 (40.8) | 4,014 (34.2) |  |  | 772 (42.5) | 834 (35.0) |  |
| ≥75 | 3,372 (30.1) | 6,094 (51.9) |  |  | 368 (20.3) | 1,234 (51.7) |  |
| Men | 6,576 (58.7) | 5,695 (48.5) | 0.206 |  | 1,194 (65.8) | 1,077 (45.2) | 0.424 |
| CHA_2_DS_2_-VASc score |  |  |  |  |  |  |  |
| Mean ± SD | 3.36±1.67 | 3.86±1.65 | 0.302 |  | 2.75±1.53 | 3.61±1.59 | 0.546 |
| Median (IQR) | 3 (2-4) | 4 (3-5) |  |  | 3 (2-4) | 4 (3-5) |  |
| 0-1 | 1,315 (11.7) | 788 (6.7) |  |  | 358 (19.7) | 203 (8.5) |  |
| 2-3 | 4,980 (44.5) | 4,191 (35.7) |  |  | 935 (51.5) | 950 (39.8) |  |
| ≥4 | 4,905 (43.8) | 6,770 (57.6) |  |  | 522 (28.8) | 1,232 (51.7) |  |
| CHADS_2_ score |  |  |  |  |  |  |  |
| Mean ± SD | 1.81±1.20 | 2.04±1.22 | 0.189 |  | 1.42±1.08 | 1.79±1.19 | 0.324 |
| Median (IQR) | 2 (1-2) | 2 (1-3) |  |  | 1 (1-2) | 2 (1-2) |  |
| Hypertension | 8,112 (72.4) | 8,628 (73.4) | 0.022 |  | 1,250 (68.9) | 1,574 (66) | 0.061 |
| Diabetes mellitus | 2,683 (24.0) | 2,732 (23.3) | 0.210 |  | 379 (20.9) | 466 (19.5) | 0.033 |
| Dyslipidemia | 4,802 (42.9) | 4,809 (40.9) | 0.039 |  | 777 (42.8) | 883 (37.0) | 0.118 |
| Heart failure | 3,501 (31.3) | 3,819 (32.5) | 0.026 |  | 358 (19.7) | 590 (24.7) | 0.120 |
| Prior MI | 354 (3.2) | 421 (3.6) | 0.023 |  | 37 (2.0) | 60 (2.5) | 0.032 |
| PAD | 1,954 (17.5) | 2,155 (18.3) | 0.023 |  | 281 (15.5) | 429 (18.0) | 0.067 |
| COPD | 2,064 (18.4) | 2,554 (21.7) | 0.082 |  | 262 (14.4) | 486 (20.4) | 0.157 |

Categorical variables, n (%)

Abbreviation: ASD, absolute standardized difference; COPD, chronic obstructive pulmonary disease; IQR, interquartile range; MI, myocardial infarction; PAD, peripheral artery disease; SD, standard deviation.

**Table S4. Baseline characteristics of propensity matched population by each dose regimens**

|  | Standard dose | | |  | Reduced dose | | |
| --- | --- | --- | --- | --- | --- | --- | --- |
|  | Rivaroxaban  (n=5,445) | Edoxaban  (n=1,815) | ASD |  | Rivaroxaban  (n=6,924) | Edoxaban  (n=2,308) | ASD |
| Age, years |  |  |  |  |  |  |  |
| Mean ± SD | 67.2±9.8 | 66.7±9.6 | 0.047 |  | 74.3±8.9 | 74.0±9.1 | 0.034 |
| Median (IQR) | 68 (61-74) | 68 (61-74) |  |  | 75 (69-80) | 75 (69-80) |  |
| <65 | 1,975 (36.3) | 675 (37.2) |  |  | 875 (12.6) | 302 (13.1) |  |
| 65-74 | 2,267 (41.6) | 772 (42.5) |  |  | 2,355 (34.0) | 798 (34.6) |  |
| ≥75 | 1,203 (22.1) | 368 (20.3) |  |  | 3,694 (53.4) | 1,208 (52.3) |  |
| Men | 3,565 (65.5) | 1,194 (65.8) |  |  | 3,205 (46.3) | 1,076 (46.6) |  |
| CHA_2_DS_2_-VASc score |  |  |  |  |  |  |  |
| Mean ± SD | 2.75±1.50 | 2.75±1.53 | 0.003 |  | 3.67±1.59 | 3.65±1.60 | 0.014 |
| Median (IQR) | 3 (2-4) | 3 (2-4) |  |  | 4 (3-5) | 4 (3-5) |  |
| 0-1 | 1,044 (19.2) | 358 (19.7) |  |  | 557 (8.0) | 191 (8.3) |  |
| 2-3 | 2,866 (52.6) | 935 (51.5) |  |  | 2,602 (37.6) | 891 (38.6) |  |
| ≥4 | 1,535 (28.2) | 522 (28.8) |  |  | 3,765 (54.4) | 1,226 (53.1) |  |
| CHADS_2_ score |  |  |  |  |  |  |  |
| Mean ± SD | 1.48±1.08 | 1.42±1.08 | 0.052 |  | 1.86±1.17 | 1.84±1.18 | 0.023 |
| Median (IQR) | 1 (1-2) | 1 (1-2) |  |  | 2 (1-3) | 2 (1-3) |  |
| Hypertension | 3,861 (70.9) | 1,250 (68.9) | 0.044 |  | 4,771 (68.9) | 1574 (68.2) | 0.015 |
| Diabetes mellitus | 1,092 (20.1) | 379 (20.9) | 0.020 |  | 1,385 (20) | 466 (20.2) | 0.005 |
| Dyslipidemia | 2,244 (41.2) | 777 (42.8) | 0.032 |  | 2,435 (35.2) | 877 (38.0) | 0.059 |
| Heart failure | 1,054 (19.4) | 358 (19.7) | 0.009 |  | 1,792 (25.9) | 590 (25.6) | 0.007 |
| Prior MI | 86 (1.6) | 37 (2.0) | 0.034 |  | 165 (2.4) | 60 (2.6) | 0.014 |
| PAD | 728 (13.4) | 281 (15.5) | 0.060 |  | 1,212 (17.5) | 416 (18.0) | 0.014 |
| COPD | 688 (12.6) | 262 (14.4) | 0.053 |  | 1,324 (19.1) | 476 (20.6) | 0.038 |

Categorical variables, n (%)

Abbreviation: ASD, absolute standardized difference; COPD, chronic obstructive pulmonary disease; IQR, interquartile range; MI, myocardial infarction; PAD, peripheral artery disease; SD, standard deviation.

**Table S5. Number of events, crude event rates according to subgroups**

| Subgroup category | Treatment group | Total number | Ischemic stroke | | ICH | | Hospitalization for GI bleeding | | Hospitalization for major bleeding | | All-cause death | | Ischemic stroke + ICH + all-cause death | |
| --- | --- | --- | --- | --- | --- | --- | --- | --- | --- | --- | --- | --- | --- | --- |
|  |  |  | Events | IR* | Events | IR* | Events | IR* | Events | IR* | Events | IR* | Events | IR* |
| Age (years) | | | | | | | | | | | | | | |
| <65 | Rivaroxaban | 2850 | 22 | 1.17 | 9 | 0.48 | 7 | 0.37 | 16 | 0.85 | 36 | 1.91 | 62 | 3.31 |
|  | Edoxaban | 977 | 5 | 1.69 | 2 | 0.68 | 1 | 0.34 | 3 | 1.01 | 5 | 1.69 | 10 | 3.38 |
| 65-74 | Rivaroxaban | 4622 | 75 | 2.37 | 29 | 0.91 | 50 | 1.57 | 79 | 2.49 | 120 | 3.75 | 203 | 6.42 |
|  | Edoxaban | 1570 | 9 | 1.93 | 4 | 0.86 | 3 | 0.64 | 7 | 1.50 | 17 | 3.64 | 29 | 6.24 |
| ≥75 | Rivaroxaban | 4897 | 129 | 4.12 | 42 | 1.33 | 95 | 3.03 | 133 | 4.26 | 334 | 10.5 | 457 | 14.7 |
|  | Edoxaban | 1576 | 24 | 5.19 | 4 | 0.86 | 16 | 3.45 | 20 | 4.32 | 53 | 11.4 | 76 | 16.4 |
| Sex | | | | | | | | | | | | | | |
| Male | Rivaroxaban | 6770 | 106 | 2.39 | 46 | 1.03 | 63 | 1.41 | 108 | 2.43 | 269 | 6.00 | 389 | 8.78 |
|  | Edoxaban | 2270 | 19 | 2.87 | 4 | 0.60 | 13 | 1.96 | 17 | 2.56 | 40 | 6.01 | 60 | 9.06 |
| Female | Rivaroxaban | 5599 | 120 | 3.21 | 34 | 0.90 | 89 | 2.38 | 120 | 3.22 | 221 | 5.85 | 333 | 8.94 |
|  | Edoxaban | 1853 | 19 | 3.38 | 6 | 1.07 | 7 | 1.24 | 13 | 2.32 | 35 | 6.20 | 55 | 9.82 |
| CHA_2_DS_2_-VASc score | | | | | | | | | | | | | | |
| 0-2 | Rivaroxaban | 4128 | 36 | 1.39 | 15 | 0.58 | 20 | 0.77 | 35 | 1.35 | 91 | 3.50 | 131 | 5.07 |
|  | Edoxaban | 1397 | 8 | 2.03 | 4 | 1.01 | 5 | 1.27 | 9 | 2.28 | 14 | 3.54 | 25 | 6.34 |
| ≥3 | Rivaroxaban | 8241 | 190 | 3.40 | 65 | 1.15 | 132 | 2.35 | 193 | 3.45 | 399 | 7.05 | 591 | 10.6 |
|  | Edoxaban | 2726 | 30 | 3.62 | 6 | 0.72 | 15 | 1.80 | 21 | 2.53 | 61 | 7.31 | 90 | 10.9 |
| Renal function (CrCl, mL/min) | | | | | | | | | | | | | | |
| ≤50 | Rivaroxaban | 506 | 16 | 4.66 | 5 | 1.44 | 9 | 2.60 | 14 | 4.07 | 31 | 8.88 | 47 | 13.7 |
|  | Edoxaban | 148 | 2 | 4.95 | 0 | 0 | 2 | 5.02 | 2 | 5.02 | 4 | 9.85 | 5 | 12.4 |
| >50 | Rivaroxaban | 9241 | 148 | 2.42 | 54 | 0.88 | 96 | 1.56 | 148 | 2.42 | 270 | 4.37 | 430 | 7.04 |
|  | Edoxaban | 3140 | 23 | 2.46 | 8 | 0.85 | 8 | 0.85 | 16 | 1.71 | 36 | 3.84 | 65 | 6.97 |
| Body weight (kg) | | | | | | | | | | | | | | |
| ≤60 | Rivaroxaban | 3767 | 84 | 3.36 | 31. | 1.23 | 59 | 2.36 | 89 | 3.57 | 169 | 6.69 | 252 | 10.1 |
|  | Edoxaban | 1255 | 16 | 4.34 | 5 | 135 | 5 | 1.35 | 10 | 2.71 | 26 | 7.01 | 44 | 11.9 |
| >60 | Rivaroxaban | 5980 | 80 | 2.01 | 28 | 0.70 | 46 | 1.15 | 73 | 1.84 | 132 | 3.29 | 225 | 5.68 |
|  | Edoxaban | 2033 | 9 | 1.48 | 3 | 0.49 | 5 | 0.82 | 8 | 1.32 | 14 | 2.30 | 26 | 4.29 |

*IR, per 100 person-years.

Abbreviation: CrCl, creatinine clearance; GI, gastrointestinal; ICH, intracranial hemorrhage; IR, incidence rate.

**II. Supplementary Figures**

**Figure S1. Distribution of propensity scores in rivaroxaban and edoxaban group before and after matching**

**
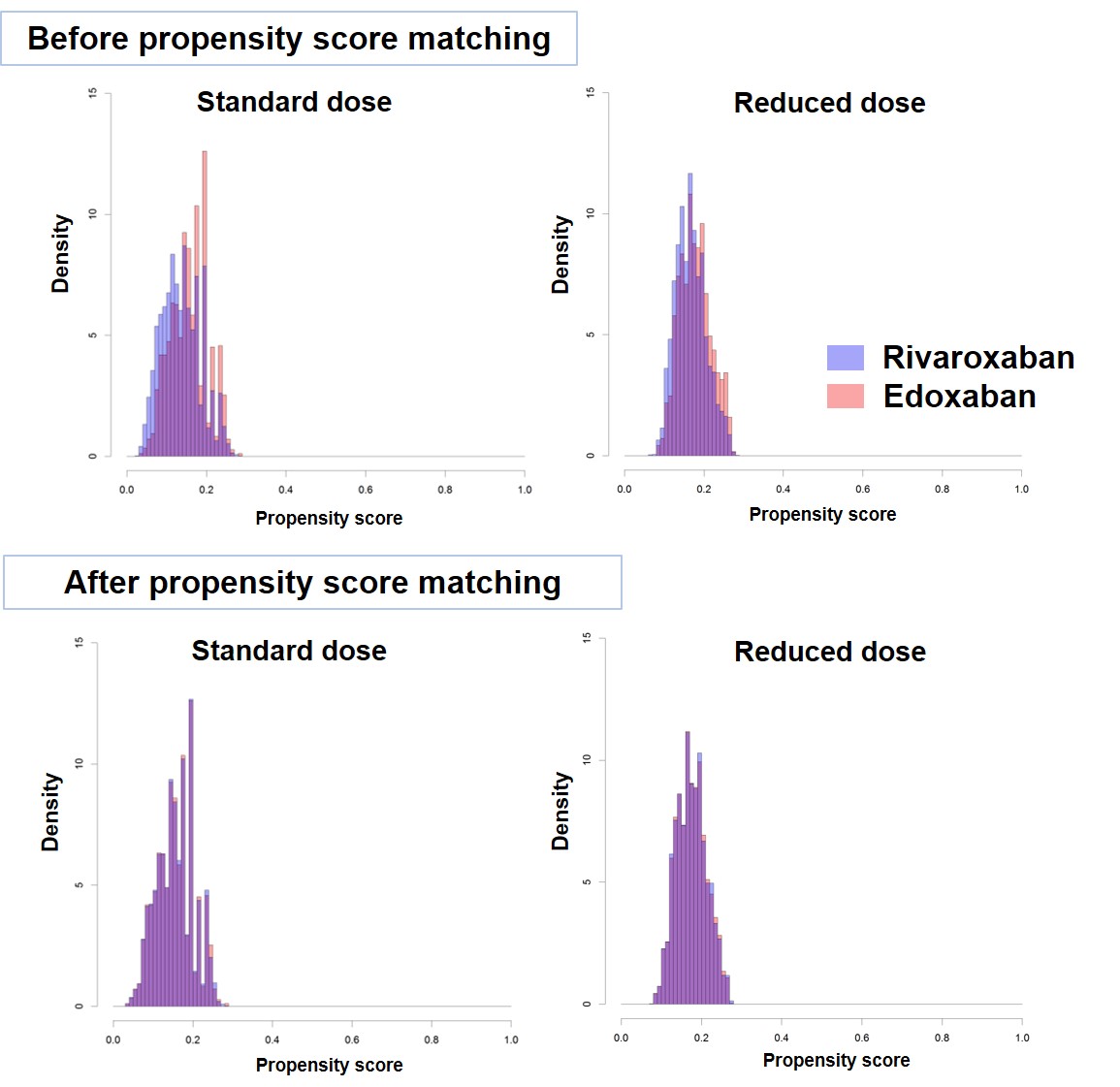
**

**Figure S2. Cumulative incidence of six clinical outcomes in rivaroxaban 20 mg and edoxaban 60 mg groups**

**
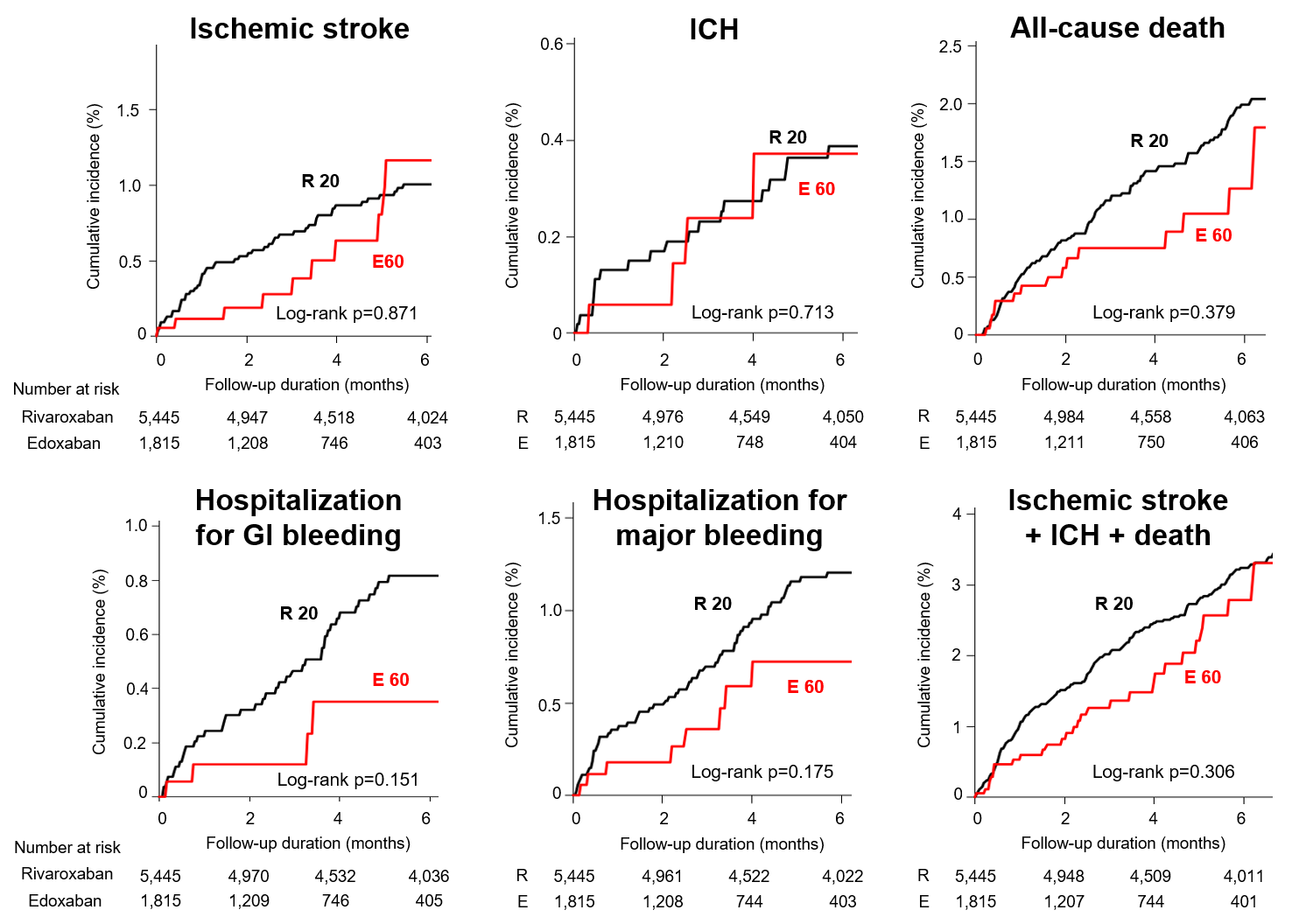
**

**Figure S3. Cumulative incidence of six clinical outcomes in rivaroxaban 15/10 mg and edoxaban 30 mg groups**

**
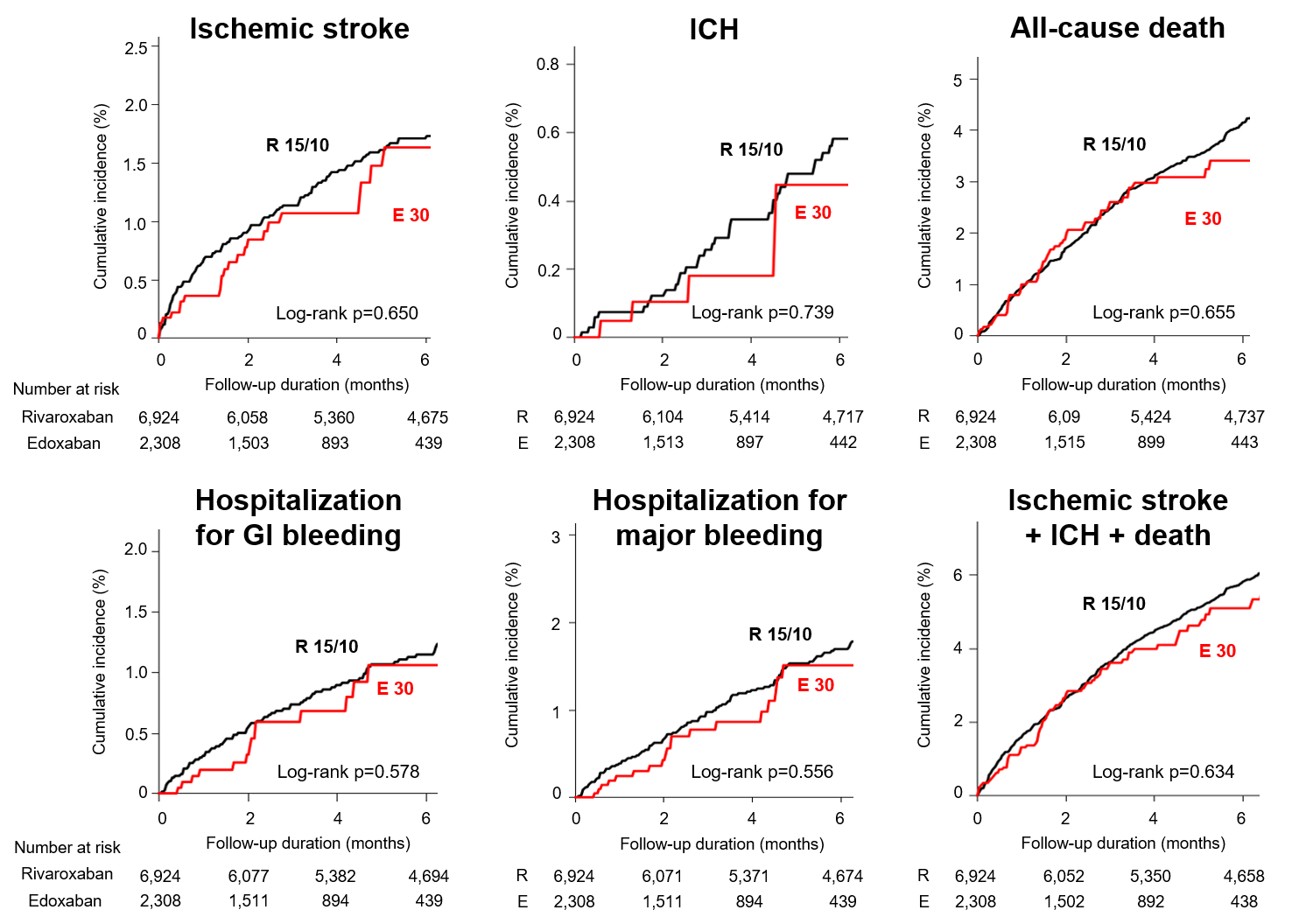
**
